# Structure-Function Network Mapping and Its Assessment via Persistent Homology

**DOI:** 10.1371/journal.pcbi.1005325

**Published:** 2017-01-03

**Authors:** Hualou Liang, Hongbin Wang

**Affiliations:** 1 School of Biomedical Engineering, Science & Health Systems, Drexel University, Philadelphia, PA, United States of America; 2 Center for Biomedical Informatics, Texas A&M University Health Science Center, Houston, TX, United States of America; University of Pennsylvania, UNITED STATES

## Abstract

Understanding the relationship between brain structure and function is a fundamental problem in network neuroscience. This work deals with the general method of structure-function mapping at the whole-brain level. We formulate the problem as a topological mapping of structure-function connectivity via matrix function, and find a stable solution by exploiting a regularization procedure to cope with large matrices. We introduce a novel measure of network similarity based on persistent homology for assessing the quality of the network mapping, which enables a detailed comparison of network topological changes across all possible thresholds, rather than just at a single, arbitrary threshold that may not be optimal. We demonstrate that our approach can uncover the direct and indirect structural paths for predicting functional connectivity, and our network similarity measure outperforms other currently available methods. We systematically validate our approach with (1) a comparison of regularized vs. non-regularized procedures, (2) a null model of the degree-preserving random rewired structural matrix, (3) different network types (binary vs. weighted matrices), and (4) different brain parcellation schemes (low vs. high resolutions). Finally, we evaluate the scalability of our method with relatively large matrices (2514x2514) of structural and functional connectivity obtained from 12 healthy human subjects measured non-invasively while at rest. Our results reveal a nonlinear structure-function relationship, suggesting that the resting-state functional connectivity depends on direct structural connections, as well as relatively parsimonious indirect connections via polysynaptic pathways.

## Author Summary

One of the major challenges in neuroscience is to understand how brain structure is related to function. In this work, we present a whole-brain method to quantify the structure-function relationship. Our data-driven approach allows the inferred functional connectivity matrix to be represented as a weighted sum of the powers of the structural matrix, containing both direct and indirect pathways. We further introduce a novel measure of network similarity based on persistent homology for assessing the goodness of fit for the mapping; such a measure enables the complete comparison of network topological changes across all possible thresholds, and thus effectively circumvents the problem of selecting the arbitrary threshold for the resulting functional networks. Our results show that our approach is able to uncover both direct and indirect structural paths for predicting functional connectivity in all three connectivity datasets, suggesting that the resting-state functional connectivity is at least in part mediated by indirect pathways, in addition to direct structural connections. The finding of a nonlinear relationship between brain structure and function is conceptually new, thus advances our understanding of how structural networks shape functional networks. This work demonstrates the potential utility of our approach in a rapidly growing field of network neuroscience.

## Introduction

The last decade has witnessed considerable progress towards understanding how the brain structural connections constrain and enable brain functional networks and the resulting behavior [1–3]. Structural connectivity (SC) refers to the ‘hard-wired’ white matter anatomic connections between brain areas and can be constructed with current noninvasive neuroimaging technologies, such as diffusion tensor imaging (DTI) and diffusion spectral imaging (DSI) [4], whereas functional connectivity (FC) generally refers to the temporal correlation between nodal activities observed in functional neuroimaging data such as functional magnetic resonance imaging (fMRI) blood oxygenation level–dependent (BOLD) signals that are acquired during task performance or the resting state [5]. In particular, resting-state fMRI has become an important basis for functional network analysis, after the discovery of intrinsic spatially distributed low-frequency fluctuations of BOLD signals [6]. Structural and functional networks each provide different, yet complementary information about brain organization and function. There is no simple relationship between structure and function. For example, brain areas not directly connected by structural links can be functionally connected [7]. Therefore, the question of how diverse functional networks arise from a relatively fixed structural architecture remains unanswered in neuroscience.

Several attempts have been made to relate the brain structure to function. One is to simply calculate the SC-FC correlation [8–13] or compare the graph theory based topological features between structural and functional networks [14–18]. An emerging approach is to use large-scale computational modeling to understand the link between structural and functional brain connectivity [19–22]. Alternative methods focus on directly performing the topological mapping of structure-function connectivity using the regression framework [23–24]. These methods are either largely descriptive, or limited to relatively small connectivity matrices and therefore difficult to build realistic whole-brain models. It remains a challenge to fully elucidate the intricate relationship between brain structure and function.

In this paper, we present a whole-brain data-driven approach to the structure-function mapping. We show that the predicted functional matrix can be represented as a weighted sum of the powers of the structural matrix, consisting of both direct and indirect effects along varying paths. We find a stable solution by exploiting the regularization procedure to cope with large matrices. We further introduce a novel measure of network similarity based on persistent homology [25–27] for assessing the goodness of fit for the mapping; such a measure enables the complete comparison of network topological changes across all possible thresholds, thereby effectively circumvents the problem of arbitrarily selecting the threshold on edge weights of the resulting functional networks.

On three connectivity datasets, we demonstrate that our approach can fully uncover the direct from the indirect structural paths in predicting functional connectivity. We examine the structure-function relationship systematically by comparing (1) the regularized vs. non-regularized procedures, (2) a null model of the degree-preserving random rewired vs. original structural matrices, (3) binary vs. weighted network types, and (4) low- vs. high- resolution parcellation schemes. Finally, we evaluate the scalability of our method with relatively large matrices (2514x2514) of structural and functional connectivity obtained from 12 healthy human subjects measured non-invasively while at rest [28]. Our results indicate a nonlinear structure-function relationship, suggesting that the resting-state functional connectivity is mainly mediated by direct structural connections, and is also contributed by relatively parsimonious indirect connections via polysynaptic pathways.

## Materials and Methods

### Low- and High-Resolution Connectivity Datasets

Structural connectivity from five healthy right-handed male participants was obtained using diffusion spectrum imaging (DSI) data and tractography [7, 14]. The gray matter was partitioned into 66 cortical regions according to anatomical landmarks, and further subdivided into 998 regions-of-interest (ROIs). The 998 ROIs were chosen to provide approximately equal size such that their boundaries aligned with those of the 66 cortical regions [14]. As a result, two different parcellations were generated in the same dataset: the low-resolution of 66 regions and the high-resolution of 998 ROIs. White matter tractography was used to estimate the fiber tract density connecting each pair of ROIs, averaged across subjects. The dataset are available from the open source Connectome mapper [29].

**Fig 1:**
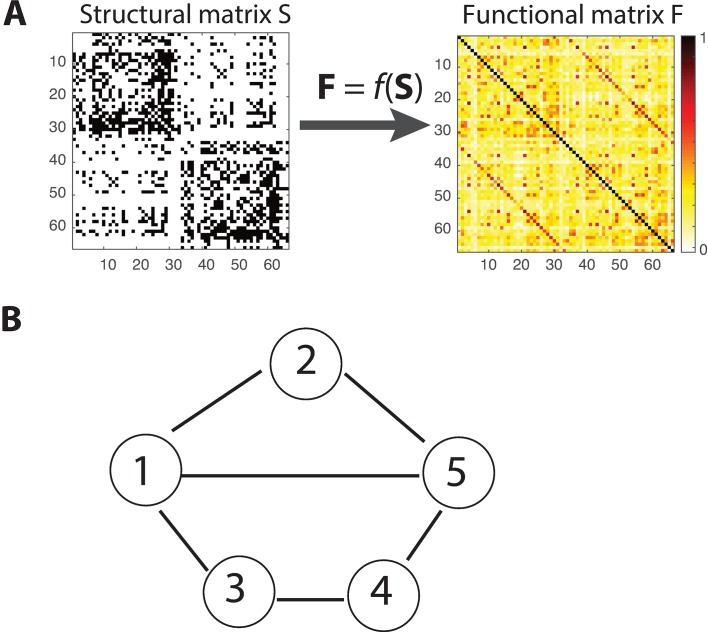
Schematic of structure-to-function network mapping and a network with both direct and indirect pathways. (A). A matric function *f* maps the structural connectivity matrix S (left) to functional connectivity matrix F (right) for all 66 brain regions. (B) Functional connectivity between nodes 1 and 5 consists of both the direct effect (the middle path between 1 and 5: 1–5) and the indirect effects of different path lengths (e.g., length 2 of the upper path between 1 and 5 via 2: 1–2–5; length 3 of the lower path between 1 and 5 via 3 and 4: 1–3–4–5).

The empirical resting-state functional connectivity was also obtained for the same subjects by measuring the corresponding fMRI BOLD signal during 20 min in absence of stimulation or task. This empirical functional connectivity matrix reflects the correlation of the BOLD activity between different brain areas at rest. The correlations of BOLD activity were computed using the ROI time series that were down-sampled to the 66-region map by averaging across all ROIs within each region. Fig 1A shows the low-resolution anatomical and resting-state functional connectivity matrices (66 x 66). Each row or column of the matrix shows a region of interest or ROI of the cortex, and its entries represent structural or functional connectivity with all the other ROIs. The matrices represent both the left and right hemispheres, with the top half of the rows/columns representing one hemisphere and the bottom half the other.

### Very Large Connectivity Dataset

The structural and functional connectivity were extracted from same-subject diffusion tensor imaging (DTI) and fMRI data acquired non-invasively from 12 healthy human subjects while at rest. It consists of a parcellation of 2514 ROIs; the dataset can be downloaded from the Brain Hierarchical Atlas NITRC page (https://www.nitrc.org/frs/?group_id=964). Further details are available in [28]. These are relatively large matrices (2514 x 2514) gathered from the same subjects.

### Structure-Function Network Mapping

Given the structural and functional connectivity matrices **S** ∈ ℝ^*N*×*N*^ and **F** ∈ ℝ^*N*×*N*^, with N being the total number of network nodes, our goal is to find a general matrix function *f* that maps **S** onto **F** (Fig 1A):

F=f(S) (1)

For any analytic function *f*, the mapping can be approximated via the Taylor series as a polynomial in **S** of degree at most N-1 due to the Cayley-Hamilton theorem [30, 31]:

$$\hat{F}=\sum_{k=0}^{N-1}c_{k}S^{k}$$ (2)

where the first term (*k* = 0) is an offset for fitting the diagonal elements of the matrix **F**, reflecting local, recurrent self-coupling within individual nodes; the second term (*k* = 1) represents the direct contribution of **S** to **F** (e.g., the middle path between nodes 1 and 5 in Fig 1B), and other higher-order terms (*k* ≥ 2) provide indirect contributions of various path lengths (e.g., **S**^2^ term or the length 2 of the upper path between nodes 1 and 5 via the intermediate node 2: 1–2–5; **S**^3^ term or the length 3 of the lower path between 1 and 5 via 3 and 4: 1–3–4–5 in Fig 1B). For **S** being a weighted structure matrix, **S**^*k*^ contains the same path information as the binary matrix at the path length of *k*, but additionally incorporates weight information into the structure. Therefore, the observed functional matrix can be represented as a weighted sum of the powers of the structural matrix, consisting of both direct and indirect effects along varying paths. This model has an underlying assumption that the maximum absolute eigenvalue of the structural matrix is smaller than one, which can be satisfied by normalizing the coupling strength of the matrix (see below).

The coefficients in (2) are typically found with the least-squares method, yet the matrix mapping becomes numerically ill-posed and rank-deficient for very large networks. To improve the model fitting, we first introduce a global brain signal as a common shared input to all the nodes, which can be represented as a constant matrix whose values are all the same. Spontaneous BOLD signal can exhibit coherence both within discrete brain networks and over the entire brain [32]. A global signal, typically defined as signal averaged across all voxels, can induce high correlations across the brain, which is often removed to better isolate functional networks. Second, we address the rank-deficient issue by solving the Tikhonov regularized least squares problem, which is given as follows:

$$\hat{c}=\mathrm{argmin}\;{\left\|F-\sum_{k=0}^{K}c_{k}S^{k}-G\right\|}_{}^{2}+\mu {\left\|c\right\|}^{2},$$

with **c** = (*c*_0_,*c*_1_,…,*c*_*K*_)^*T*^, and the summation term is the estimated functional connectivity matrix of maximum path length K, which can be determined by the goodness of fit (See below). G is a constant matrix denoting the global shared input. The regularization parameter *μ* can be determined by the generalized cross-validation (GCV) [33, 34]. GCV provides a robust estimate of how well a given model would fare under cross-validation testing (particularly how overfit or underfit the model is) without the demanding computational burden for performing every cross-validation. We have used the GCV as described in [35]. The statistical significance of the estimated coefficients can be assessed with the bootstrap resampling procedure. Unless otherwise stated, S is a binary structural matrix (where connections are either absent or present) that is converted from the complete weighted matrix; it hence has the same number of edges as the original structural matrix.

When solving the above optimization problem, a couple of important practical issues ought to be considered. First, only the diagonal and the lower triangular part of the matrices are needed since all the matrices involved are symmetric. Second, with the increasing path length, the matrix powers of **S** rapidly blow up, which becomes a rather severe issue for large matrices. To avoid the numerical overflow of the power series, before the mapping is performed, we normalize each matrix power by dividing every element by a normalization factor, which is defined as the maximum absolute element of the matrix, i.e., **S**^*k*^/*max*(|**S**^*k*^|). We then restore the same normalization factor to the corresponding estimated coefficient to obtain the coefficients conforming to the original matrix power. As a result, the estimated coefficients at higher path lengths are typically rather small due to the large values of the higher matrix powers.

### Network Similarity Measure Using Persistent Homology

To compare the empirical and predicted functional networks, we introduce a new measure of network similarity based on persistent homology [25–27, 36]. Calculating persistent homology generally requires a measure of distance or dissimilarity between nodes. We can convert the correlation-based connectivity matrix to a dissimilarity matrix via 1– |correlation|. To fix the idea, we first construct a binary network from the dissimilarity matrix: two nodes are connected by an edge if their distance is less than a parameter *λ* (called the filtration value in persistent homology). At *λ* = 0, the number of connected components (also known as the zeroth Betti number, *β*_0_) is equal to the number of nodes, since no edge links are included. As *λ* is increased, the number of connected components declines. As a result, the *persistence* represented by *λ* measures how long node pairs, hence path-connected components, stay connected as the filtration value *λ* varies. Observing the topological evolution over changing filtration values leads to a network filtration, which allows the construction of a minimum spanning tree [37, 38].

**Fig 2:**
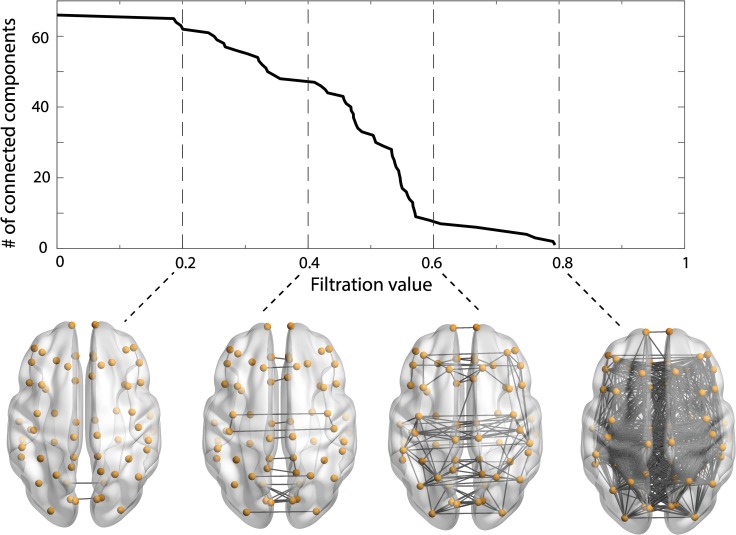
The barcode (the zeroth Betti number) for target functional network over changing filtration values (top) and the network connectivity maps at some selected filtration values (bottom). Network renderings with BrainNet Viewer [41].

The changes in connectedness can be quantified by a barcode summarizing the zeroth Betti number, which counts the number of path-connected components, across a range of *λ* [27]. The so-called barcode, as illustrated in Fig 2 (top) for the low-resolution dataset, consists of a plot tracking the fate of connected components as the filtration value *λ* changes, with connectivity maps of functional network at some selected *λ* shown at the bottom of Fig 2. We measure the goodness of fit of the mapping as the sum of squared errors (*SSE*_*β*_) between the barcodes for the target (*β*_0_(*λ*)) and predicted (${\hat{\beta }}_{0}(\lambda )$) networks, which is defined as follows:

$$SSE_{\beta }=\frac{1}{N^{2}}\int_{0}^{1}\left({\hat{\beta }}_{0}(\lambda \right)-\beta_{0}(\lambda ){)}^{2}d\lambda$$

The normalization by N^2^, where N is the total number of network nodes, renders this measure independent of the network size, thus facilitates the comparison between different networks. As the path length grows, the SSE of the mapping decreases. The maximum path length K is selected where the SSE stops descending precipitously. A key advantage of using persistent homology is that one can examine a graph filtration generated by all possible thresholds and systematically analyze the persistence of network topological changes across these thresholds [39, 40]. Therefore, our new measure is ideal for comparing brain network topologies as it does not rely on any fixed threshold, but instead encompasses all the detailed changes in network architecture in the filtration.

## Results

### Structure-Function Network Mapping at Low Resolution

**Fig 3:**
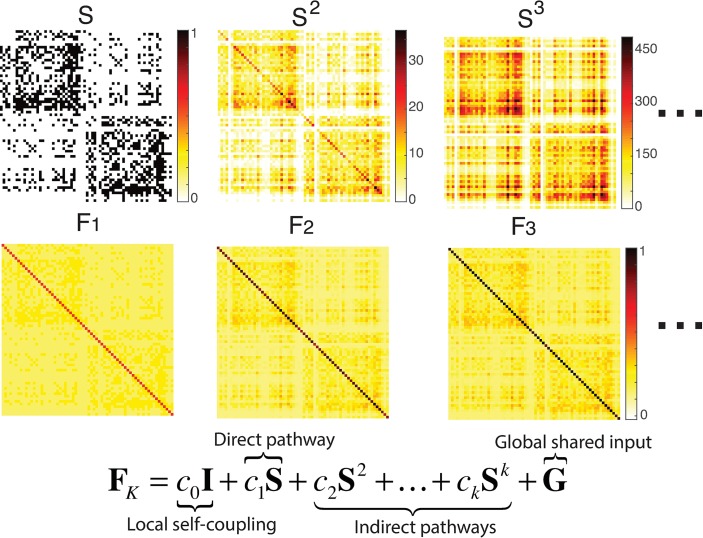
The snapshots of the structural networks successively with direct connection, length-2 and length-3 indirect connections (top row from left to right), and the inferred functional networks at maximum path length of 1, 2 and 3, respectively (middle row). The inferred functional matrix is represented as a weighted sum of the powers of the structural matrix, consisting of both direct and indirect pathways in addition to the local, recurrent self-coupling within nodes and the global shared input (bottom).

We first carried out network mapping for low-resolution connection matrices with a size of 66 x 66 (N = 66 anatomical subregions, see Methods and Fig 1A). Fig 3 in the top row provides the snapshots of the structural matrices sequentially with direct connection, length-2 and length-3 indirect connections, whereas in the middle row shows the inferred functional connectivity matrices at maximum path length of 1, 2 and 3, respectively. The inferred functional matrix is a weighted sum of the powers of the structural matrix, shown in the bottom of Fig 3, consisting of both direct and indirect pathways in addition to the local, recurrent self-coupling within nodes and the global shared input.

**Fig 4:**
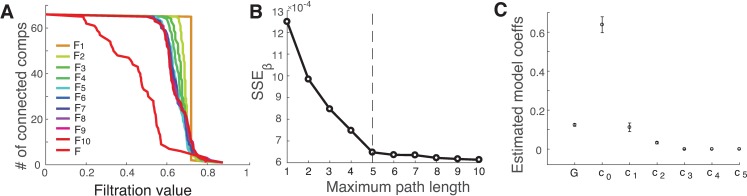
Performance of network mapping (66 regions). (A) The barcodes for the inferred functional networks and the target functional network as a function of the filtration value. (B) *SSE*_*β*_ decreases as the path length increases, and saturates at the maximum path length of 5 (the vertical dashed line). (C) The estimated global shared input and model coefficients at K = 5, with the error bars indicating the 95% confidence interval.

Next, we performed persistent homology analysis to assess the quality of the network mapping. Fig 4A shows the barcodes of the inferred functional matrix at different path lengths and the target functional connectivity matrix (red curve), where we see across the full scales that the inferred networks approach the target network as the path length increases. The goodness of fit, indexed by *SSE*_*β*_, is shown in Fig 4B, where we see the *SSE*_*β*_ decreases as the path length increases, with the maximum path length of 5 (the elbow point) containing most of the structural information needed to infer functional connectivity. Fig 4C displays the estimated global shared input and model coefficients up to K = 5, with the error bars indicating the 95% confidence interval obtained via the bootstrap procedure. We note that the coefficients alone are not a reliable indicator for determining the influence of each path length as the matrix powers at the longer path lengths can have very high values, particularly for large matrices.

#### Comparison with other network similarity measures

**Fig 5:**
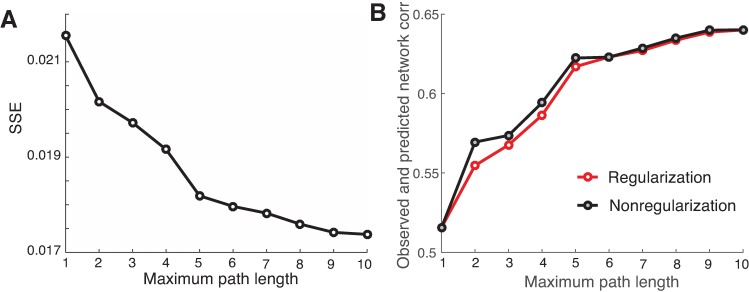
Comparison of *SSE*_*β*_ with the regular SSE (A) and the correlation between the observed and predicted functional networks (B) for both the regularized (red) and non-regularized (black) methods.

To check the sensitivity of our network similarity measure, we compared it with two commonly used methods. One is the conventional sum of squared errors (SSE) using the Frobenius norm, which is simply based on the direct difference of two matrices; another is to calculate the Pearson correlation coefficient between all corresponding pairwise elements of both matrices. The results are shown in Fig 5A and 5B (red curve), respectively. It seems that our measure is quite similar to both SSE and the correlation. Yet, it is also evident that there is a lack of clear ‘elbow’ point for determining the maximum path length for both methods, though the correlation-based method provides some indication at K = 5, albeit less conspicuous when compared to Fig 4B. These results indicate that our new measure is rather sensitive in comparing network similarity.

#### Regularization vs. non-regularization methods

As a cross validation of our regularization procedure, we compared it to the non-regularized method [24, 30] on small matrices. We implemented the non-regularized method using the non-linear regression algorithm (the MATLAB function *nlinfit*), and obtained remarkably consistent results with our method in terms of model fitting and the estimated model coefficients. As an example, we showed the correlation as a function of maximum path length in Fig 5B (black curve), which closely matched that obtained via our regularization procedure. It should be noted that, however, this is not the case when mapping large networks such as the high-resolution connection matrices (998 x 998) analyzed in the following sections, as the non-regularized method would run into the rank-deficient problem, hence unable to handle the large networks.

#### Null model analysis

**Fig 6:**
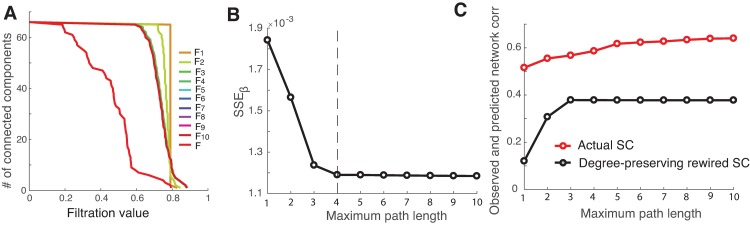
Performance of network mapping with the degree-preserving random rewired topology of structural matrix. (A) The barcodes for the inferred functional networks as a function of filtration value. (B) *SSE*_*β*_ decreases as the path length increases, and saturates at the maximum path length of 4 (the vertical dashed line). (C) Comparison of the correlation between the observed and predicted functional networks for actual SC (red) and rewired SC (black).

To assess whether our observations are directly shaped by the underlying structural network or can be explained by a random process, we consider a null model by creating random networks based on the structural network that can be used to create a benchmark value for significance test. We generate a randomized null-model network in which the degrees of all nodes are strictly preserved [42]. The results of the analysis are displayed in Fig 6, showing that the barcode for the inferred functional network at an increasing path length is not able to approach that obtained from the actual functional network (Fig 6A). Although we observe the decline in SSE for paths of length up to 4 (Fig 6B), as also reflected in the barcodes, the overall SSE values are substantially larger for the degree-preserving random rewired matrices than those otherwise not randomly rewired. This can be clearly seen by comparing the correlation between the empirical and predicted networks for the actual and rewired SCs (Fig 6C), indicating that the observed structure-function relationship can hardly be reproduced with the randomly rewired structural matrices. The data suggest that the functional connectivity is tightly dependent on the underlying structural connections.

#### Binary vs. weighted structural matrices

**Fig 7:**
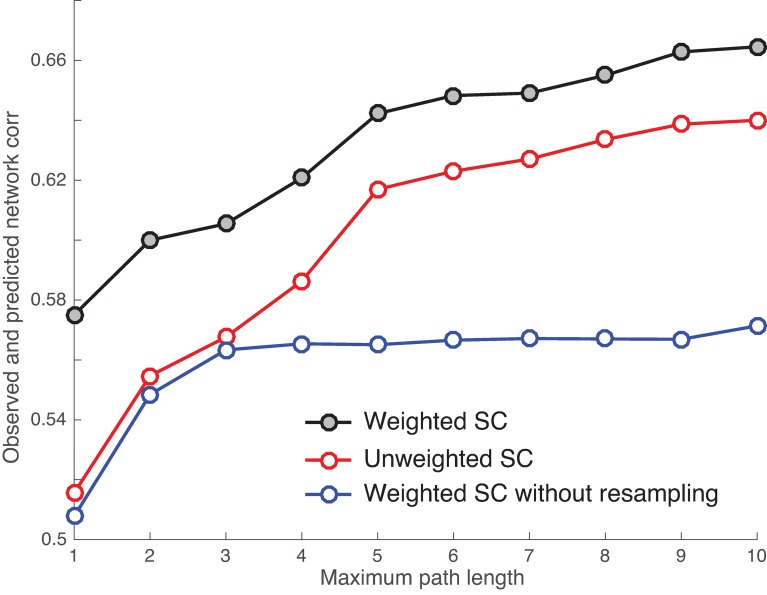
Comparison of the correlation between the observed and predicted functional networks for using binary, unweighted SC (red) with the weighted SC with (black) and without (blue) resampling in the mapping.

Thus far, we have considered binary (unweighted) structural matrix in which connections are either absent or present. This approach discards the information of fiber connection strengths available in the anatomical matrix since it is complete weighted network. To examine the impact of different network types, we compared the binary vs. weighted structural matrices on the mapping. Because the raw fiber strength data, produced by the streamline tractography, were heavily tailed, spanning about four orders of magnitude, we resampled the fiber strengths into a Gaussian distribution [7] to reduce the dynamic range. Briefly, given the raw data values, we replaced the rank-ordered the fiber count values with the same number of random samples, also rank-ordered, generated from a Gaussian distribution with a mean of 0.5 and a standard deviation of 0.1. We compared the resampled SC with the raw SC (i.e., weighted SC without resampling) as well as the binary SC in the mapping. The results are shown in Fig 7, where we observe that 1) all the correlation coefficients between actual and predicted functional matrices grow with the path length, 2) the weighted SC with resampling provides a slightly better fit than the binary SC to the functional network, yet both saturate at the same maximum path length of 5, and 3) the raw SC shows the worst performance for the mapping, with the path length up to 3 containing most of structural information for the prediction. These results indicate that the weight of links in a structural network, with the proper rescaling, can provide more information for the structure-function mapping than the binary unweighted network.

### Structure-Function Network Mapping at High Resolution

To demonstrate that our regularization method is able to handle large matrices, we scale up the mapping matrices to high-resolution parcellation (998 x 998), which is a refinement of the low-resolution (66 x 66) surface partition. Importantly, by comparing the low- and high-resolution matrices on the same dataset, we can examine the extent to which the mapping is influenced by the choice of different parcellation schemes.

**Fig 8:**
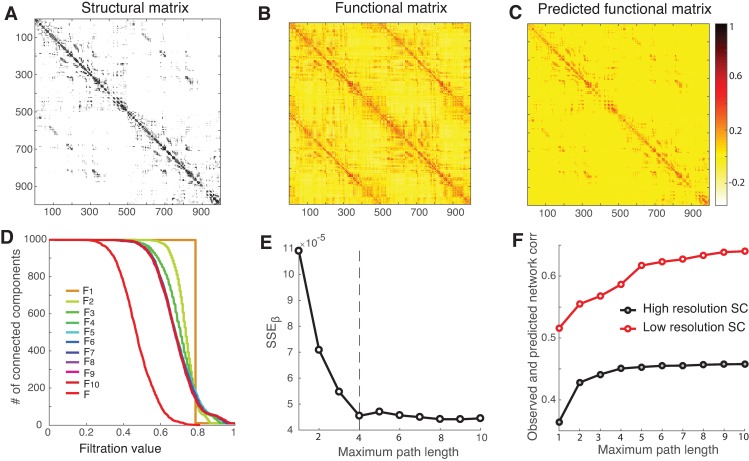
Performance of network mapping (998 ROIs). (A-C) The structural and functional networks, along with the inferred functional networks at the maximum path length of 4. (D) The barcodes for the inferred functional networks and the target functional network as a function of the filtration value. (E) *SSE*_*β*_ decreases as the path length increases, and saturates at the maximum path length of 4 (the vertical dashed line). (F) The comparison of the correlation between the observed and predicted functional networks for the low- and high-resolution structural networks.

Fig 8A and 8B display, respectively, the structural and functional connectivity matrices, with a size of 998 x 998. Fig 8C shows the predicted functional connectivity matrix at the maximum path length of 4, which is determined by the *SSE*_*β*_ curve (Fig 8E) based on the barcodes shown in Fig 8D. The estimated coefficients, together with their 95% confidence intervals, are listed in Table 1, where we see that the large weight in the direct pathway relative to the indirect paths, in addition to remarkably strong local, inhibitory self-coupling within nodes and the modest global shared input to all the nodes. Note that the differential coupling of positive and negative coefficients at different path lengths, although the exact nature is unknown, could indicate the direction of the signal change in the underlying neuronal events. The comparison of the correlation between the observed and predicted functional networks for the low- and high-resolution structural connections is shown in Fig 8F. Clearly, the anatomical connectivity is better reflected by FC at low spatial resolution and is only weakly correlated with FC at high resolution, a finding consistent with previous studies [7]. In addition, regardless of whether the parcellation is of low or high spatial resolution, we observe that the paths of length up to 5 in the brain graph contain most of the structural information needed to predict functional connectivity. This observation is striking with important implications for understanding the structure-function relationship, suggesting that the functional interactions are mediated by rather parsimonious polysynaptic anatomical connections.

**Table 1:** The estimated global shared input and model coefficients and their 95% confidence intervals for the structure-to-function network mapping at the path length of 4 for the dataset of 998 ROIs.

	Estimated coefficients	95% confidence interval
G	-0.0113	[-0.0116–0.011]
*c*_0_	-0.336	[-0.35–0.323]
*c*_1_	0.0457	[0.042 0.0494]
*c*_2_	0.00954	[0.00914 0.00994]
*c*_3_	0.000348	[0.000329 0.000366]
*c*_4_	-8.32E-06	[-8.62E-06–8.02E-06]

### Structure-Function Network Mapping at Very High Resolution

**Fig 9:**
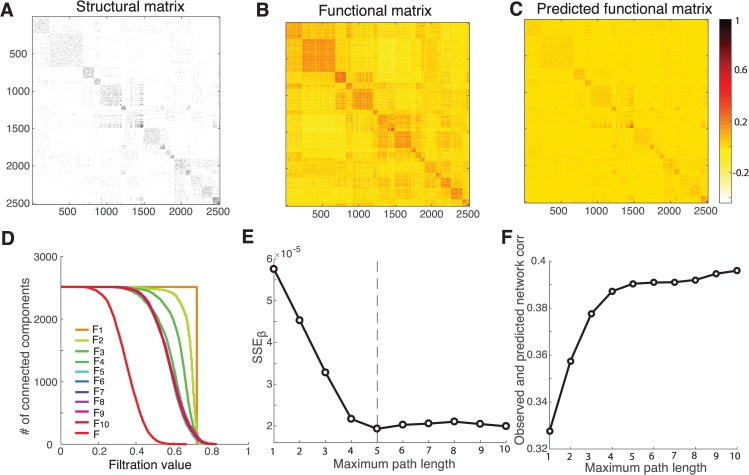
Performance of network mapping (2514 ROIs). (A-C) The structural and functional networks, along with the inferred functional networks at the maximum path length of 5. (D) The barcodes for the inferred functional networks and the target functional network as a function of the filtration value. (E) *SSE*_*β*_ decreases as the path length increases, and saturates at the maximum path length of 5 (the vertical dashed line). (F) The correlation between the observed and predicted functional networks grows as the path length increases, and gets stabilized at the maximum path length of 5.

As a final test, we evaluate the generalizability and scalability of our method using a new dataset consisting of relatively large matrices (2514x2514) of structural and functional connectivity obtained from 12 healthy human subjects measured non-invasively while at rest. Fig 9A–9C, respectively, show the structural connection, functional connectivity matrices and the predicted functional matrix at the maximum path length of 5, as determined by the goodness of fit shown in Fig 9E.

Table 2 shows the estimated coefficients and their 95% confidence intervals. We observe very strong local self-coupling and modest global shared input. Similar to other datasets analyzed above, the direct pathway again shows much stronger influence than the indirect pathways; the influence is gradually fading with increasing path length. The barcodes derived from the persistent homology analysis for the inferred functional matrices at the path length ranging from 1 to 10 are presented in Fig 9D. Though the correlation between structural and functional matrices increases as more indirect paths are involved (Fig 9F), the overall correlation values however are rather modest, as compared to the small matrices. These results suggest that finer parcellation of anatomical connectivity may be more vulnerable to scanning noise and fiber reconstruction errors. It is intriguing to note that the structural paths of length up to 5 seem to be highly robust across various sizes of the matrices for achieving optimal prediction of functional connectivity. These results together provide the compelling evidence that resting-state functional connectivity depends on direct underlying structural connections within a relatively economical polysynaptic pathway.

**Table 2:** The estimated global shared input and model coefficients and their 95% confidence intervals for the structure-to-function network mapping at the path length of 5 for the dataset of 2514 ROIs.

	Estimated coefficients	95% confidence interval
G	0.00186	[0.00176 0.00196]
*c*_0_	-0.518	[-0.527–0.509]
*c*_1_	0.0879	[0.085 0.0908]
*c*_2_	0.0199	[0.0195 0.0203]
*c*_3_	0.000266	[0.000237 0.000295]
*c*_4_	-2.23E-05	[-2.3E-05–2.15E-05]
*c*_5_	2.22E-07	[2.16E-07 2.29E-07]

## Discussion

In this paper, we have presented a whole-brain data-driven approach to the structure-function mapping. Such a mapping allowed the inferred functional matrix to be represented as a weighted sum of the matrix powers of the structural connections, containing both direct and indirect pathways. This representation unveils a nonlinear relationship between the underlying structural connections and the observed functional networks, as confirmed by all three connectivity datasets used in this study. We have further introduced a novel measure of network similarity based on persistent homology for assessing the quality of the network mapping; such a measure enabled the complete comparison of network topological changes across all possible thresholds, and thus effectively circumvented the problem of selecting the arbitrary threshold for the resulting functional networks. We demonstrated that our approach could uncover the direct as well as the indirect structural paths in predicting functional connectivity.

We provided a more detailed characterization of our approach in four different aspects. First, we compared our regularized verse the non-regularized procedures. For small matrices, both methods yielded consistent results, which provided a validation for our regularization procedure. The non-regularized method, however, failed for large matrices such as 998 x 998 due to rank deficiency problems. In essence, our whole-brain structure-function network mapping is just to solve a large-scale optimization problem with Tikhonov regularization, also known as *l*_2_ regularization, which perhaps is the most commonly used method of regularization of ill-posed problems. There is a rich body of literature on sparse network models such as compressed sensing, graphical-LASSO, sparse partial correlations [43–49]. It is conceivable that such regularization can be replaced with the *l*_1_ regularization or LASSO [43] in the objective function. Unlike the Tikhonov regularization, the LASSO can set some coefficients to zero. As such, the interpretations can be different, which would require some additional justifications relative to the path length.

Second, we performed a null model analysis with the degree-preserving randomly rewired structural topology. The random rewiring allowed us to assess whether our observations were directly shaped by the fixed structural network or could be simply explained by a random process. We found that the observed structure-function relationship cannot be reproduced with the rewired structural matrices, suggesting that structural networks impose critical constraints on functional networks. Therefore, the null model is an important control to assay the dependence of functional networks on structural connections. Additionally, it can be used as a benchmark value for statistical significance test. The null model has been increasingly used to assess what network features are important in separating different types of networks [50, 51]. The best way to generate random networks, however, is still an actively debating topic for brain network studies [52, 53]. Determining what topological information in the structural data is driving the structure-function network mapping will be important for understanding the nature and relationships between networks. Although we have used a null model in which the degrees of all nodes are strictly preserved, other null models such as a minimally wired network [63] are possible. Further studies are needed in terms of the more biologically meaningful null models as benchmarks.

Third, we examined the impact of different network types (binary vs. weighted matrices) on the mapping. The key observation was that the weighted structural network seemed always to provide more information for the structure-function mapping than the binary unweighted network, with the caveat of proper weight rescaling. The rescaling was due to the fiber counts straddling several orders of magnitude; it was not realistic to have such a large range for inter-areal physiological efficacies [7]. As we observed, the model did not perform well on the raw fiber data, especially for large matrices where the model coefficients rapidly blew up with the increasing path length. Thus, it is crucial to resample the fiber values into a Gaussian distribution to reduce the their large range before the mapping is performed or simply use the binary structural matrix.

Fourth, we determined how the mapping was influenced by different parcellations of brain regions by comparing the fine and coarse structural matrices, i.e., high and low spatial resolutions, on the same dataset. In general, we observed that (1) for either low- or high-resolution parcellation, the structure-function correlation grows with the increasing paths of length up to 5, the direct structural path of which has the strongest influence on the resulting functional connectivity, and (2) the overall structure-function correlation values are somewhat modest for large matrices, as compared to the small matrices. Our observation seems rather general, albeit relatively small sample size, as confirmed for large matrices (2514 x 2514) from a different dataset of 12 subjects. This analysis suggests that the resting-state functional connectivity is primarily mediated by direct structural connections, with incremental contributions from relatively short indirect pathways up to length 5 in the underlying structural connections.

The elbow-point selected at the path length 4–5 in our analysis is visually determined. The choice of an optimal path length K is often ambiguous, with interpretations depending on the data. Knowing the exact number of path length is desirable, but does not add fundamentally to our main conclusion that the resting-state functional connectivity is in part dependent on direct structural connections, but also indirect connections via polysynaptic pathways. Nonetheless, examining how much correlation explained by each path length may offer a hint to the relative importance of structural paths of different length in the mapping. For example (Fig 5B), at the path length of 1, there is a correlation of 0.516 when only direct structural path is considered. The correlation jumps up to 0.555, which is 3.9% increase of correlation (i.e., 0.555–0.516 = 0.039) when structural path lengths of 2 are considered. As we consecutively add structural path length of 3 to 7 into the mapping, the correlation increase at each corresponding path length are, respectively, 1.3%, 1.9%, 3.0%, 0.6%, and 0.4%. Notice how the incremental improvement is gained by including each path length, becoming negligible for *K* ≥ 5. It is also worth mentioning that the path length up to 5 does not necessarily related directly to the diameter of the structural network [2]; the maximum path length is generally shorter than the network diameter (e.g., the diameter is 6 for 998-node network, and is 9 for the 2524-node network). Nonetheless, it seems that the diameter of the structural brain network provides a much tighter upper bound than the network size for the length of the paths used in the Taylor-series approximation (Eq. 2). We also note that the overall low structure-function correlation observed in the finer parcellation of anatomical connectivity, which could be due to (1) the noise potentially introduced in the scanning, and (2) the low quality of structural connectivity for long interhemispheric fiber tracts through the corpus callosum, which were not detected because of the limited resolution of the imaging/tractography techniques [14].

The structure-function topological mapping introduced in this study is a general method developed within the framework of matrix function [31]. To determine the coefficients in the model (Eq. 2), there are several options that can be deemed as the special cases. One option is to simply consider the coefficients as some specific constants so that a closed-form solution can be achieved. For example, when *c*_*k*_ = 1, we have **F** ≈ 1/(**1**−**S**), which forms the basis of network deconvolution algorithm [54]. Similarly, when *c*_*k*_ = 1/*k*!, we have $F=\sum_{k=0}^{N-1}\frac{S^{k}}{k!}\approx e^{S}$, whereby the k! in the denominator indicating that longer paths contribute disproportionately less compared to shorter paths. Such a closed-form solution leads to the well-known measure of network communicability [55]. In this work, we have focused upon mapping structural connections to functional networks. Similarly, we can infer functional connectivity from structural connections [56] by simply inversing the role of **F** and **S**. It can be done from the algorithmic viewpoint [24], but anatomical verification is preferred. In addition, unlike the structure-to-function mapping, the interpretation of the inverse mapping becomes less straightforward.

Persistent homology is a method used in topological data analysis to characterize topological structures such as connectedness and holes in high-dimensional data [25–27]. Traditionally, it has been applied to point-cloud data, though recently it has become increasingly prominent in network neuroscience to uncover the topological structure of data [36–39, 57–62]. A unique feature of persistent homology is that it allows one to examine the changes in network architecture over a full spectrum of possible thresholds rather than just at a single fixed threshold value. To assess the quality of the network mapping, we analyzed the topological structures of these inferred functional networks against the target empirical functional network, and examined the persistent homology by calculating the zeroth Betti number (*β*_0_), i.e., the number of connected components. Earlier work has showed that, *β*_0_, the persistence of components, can be used to classify pediatric attention deficit hyperactivity disorder (ADHD), autism spectrum disorder (ASD) and control subjects [38]. Similarly, we found that our *β*_0_-based network similarity measure was sensitive enough to capture the changes in network topology with different path lengths, and compared favorably to other existing methods. Though our measure is based only on the zeroth Betti number, it is important to note that other Betti numbers such as *β*_1_ (the first Betti number) and *β*_2_ (the second Betti number), corresponding to topological circles and trapped volumes in the data, respectively, capture other higher-order homologies in the data and therefore can also be used to measure more complex structures of the network. As recently demonstrated [63], relative persistent clique patterns can be identified in the brain via higher-order Betti numbers. We note that, however, the interpretation of weighted network architecture via persistent homology does not in itself account for the presence of spurious network links which can be characterized via appropriate null networks.

When measuring the functional connectivity, we have used the Pearson correlation coefficient. Other measures such as partial correlation or Granger causality can also be used [64–66]. For example, partial least squares method has been recently used to investigate the association between structural networks and functional networks [67]. Likewise, conditional Granger causality, which statistically removes the common input and the indirect influences between a given ROI pair, has been shown to be more appropriate to quantify the strength of the functional interaction enabled by the fibers linking the a pair of ROIs [13]. A detailed comparison at the whole-brain scale of each measure’s performance against well-characterized connectivity data would serve to identify their relative strengths and weaknesses.
